# A single-blind, dose-escalation, phase I study of high-fluence light-emitting diode-red light on Caucasian non-Hispanic skin: study protocol for a randomized controlled trial

**DOI:** 10.1186/s13063-019-3278-7

**Published:** 2019-03-20

**Authors:** Erica B. Wang, Ramanjot Kaur, Julie Nguyen, Derek Ho, Evan Austin, Emanual Maverakis, Chin-Shang Li, Samuel T. Hwang, R. Rivkah Isseroff, Jared Jagdeo

**Affiliations:** 1 Department of Dermatology, Stanford University, 269 Campus Drive CCSR 2150, Stanford, CA 94305 USA; 2 Dermatology Service, Sacramento VA Medical Center, 10535 Hospital Way, Mather, CA 95655 USA; 3 Department of Dermatology, University of California Davis, 3301 C Street, Suite 1400, Sacramento, CA 95816 USA; 4 Department of Dermatology, The State University of New York Downstate Medical Center, 450 Clarkson Avenue MSC 46, Brooklyn, NY 11203 USA; 5 School of Nursing, The State University of New York, University of Buffalo, 3435 Main St, Buffalo, NY 14214 USA; 6 Dermatology Service, VA New York Harbor Healthcare System – Brooklyn Campus, 800 Poly Pl, Brooklyn, NY 11209 USA

**Keywords:** Light-emitting diode-red light, Visible red light, High fluence, Phototherapy, Study protocol, Safety, Phase I, Dose escalation, Randomized controlled trial

## Abstract

**Background:**

Visible light (400 to 700 nm) is common in our environment, comprising 44% of total solar radiation and a large component of environmental light exposure. The effects of visible light on skin remain undefined. The red light portion of the visible spectrum (600 to 700 nm) may be used to treat skin diseases as a monotherapeutic modality or in combination with other agents. Light-emitting diode-red light (LED-RL) phototherapy may represent an important advance in light-based treatment modalities because it is non-invasive, inexpensive, portable, and easily combinable with other therapies. We previously determined the maximum tolerated dose (MTD) of high-fluence LED-RL (HF-LED-RL) in skin of color individuals to be 320 J/cm^2^. To the best of our knowledge, no clinical trials have been performed to determine the safety of higher doses of HF-LED-RL in Caucasian non-Hispanic individuals. The aim of this study is to investigate the safety of HF-LED-RL at doses of 480 and 640 J/cm^2^ in healthy Caucasian non-Hispanic individuals.

**Methods:**

This is a single-blind, dose-escalation, randomized, controlled, phase I trial titled Safety Trial Assessing Red-light on Skin (STARS) 2. Healthy subjects will be randomly assigned to groups of five (three subjects randomly assigned to HF-LED-RL phototherapy and two subjects randomly assigned to mock therapy). Subjects in group 1 will receive HF-LED-RL or mock irradiation at the starting dose of 480 J/cm^2^, and the dose will be escalated in the subsequent group (group 2) to 640 J/cm^2^. The MTD is defined as the dose level below the dose at which two or more subjects (>20% of the cohort) experience a dose-limiting toxicity (DLT). After either the MTD is established or the study endpoint of 640 J/cm^2^ is achieved, additional HF-LED-RL phototherapy subjects and mock therapy subjects will be enrolled at that fluence (group 3) for a total number of up to 60 subjects. Each subject will receive a total of nine irradiation sessions, three times per week for three consecutive weeks.

**Discussion:**

This follow-up study aims to provide important knowledge about safety and cutaneous effects of HF-LED-RL phototherapy of 480 and 640 J/cm^2^ in Caucasian non-Hispanic subjects. The importance of this clinical trial is that it may establish new treatment paradigms and a safety profile for LED-RL based on race and ethnicity.

**Trial registration:**

ClinicalTrials.gov Identifier: NCT03433222. Registered on February 1, 2018 - Retrospectively registered.

**Protocol date and version:** January 12, 2018; version 1.

**Electronic supplementary material:**

The online version of this article (10.1186/s13063-019-3278-7) contains supplementary material, which is available to authorized users.

## Background

Visible light, corresponding to wavelengths 400 to 700 nm, is common in our environment, comprising 44% of total solar radiation and a large component of environmental light exposure [1, 2]. The cutaneous effects of the visible spectrum are relevant because human skin is repeatedly exposed to visible light. However, the effects of visible light on skin remain undefined. Emerging literature demonstrated that visible light irradiation from light-emitting diode (LED) or halogen incandescent light sources induces dose-dependent immediate pigmentation, immediate erythema, and delayed or sustained tanning, especially in melanocompetent individuals [3–6]. Furthermore, hyperpigmentation induced by visible light has been demonstrated to be darker and more persistent than pigmentary changes induced by long-wavelength ultraviolet (UV) A [6].

The red light portion of the visible spectrum (600 to 700 nm) may be used to treat skin diseases as a monotherapeutic modality or in combination with other agents such as photosensitizers in red light photodynamic therapy [7–9]. Low fluences (doses) of light-emitting diode-red light (LED-RL), compared with control and blue LED lights, have been shown to significantly increase wound healing, growth factor expression, and collagen fiber proliferation in a rabbit skin model [10]. Higher fluences greater than 160 J/cm^2^ per session of LED-RL (HF-LED-RL) may have the potential to treat skin fibrosis by inhibiting collagen synthesis and fibroblast proliferation and migration *in vitro* [11, 12]. Additionally, previous randomized controlled trials (RCTs) demonstrated efficacy of LED-RL for treatment of acne and facial rhytides, leading to US Food and Drug Administration (FDA)-cleared devices [13, 14]. Therefore, LED-RL has shown therapeutic benefit for some dermatologic diseases and conditions and may be of value for additional skin diseases and conditions.

LED-RL has many advantages as a therapeutic modality. An essential safety characteristic of red light, compared with UV light or other ionizing wavelengths, is that it is not known to generate DNA damage associated with skin malignancies and photo-aging [15]. Furthermore, the greater cutaneous penetration depth of visible red light up to 8 mm, compared with other wavelengths in the UV and visible light spectrum, is sufficient to reach the entire dermis [16, 17]. One beneficial aspect of LED-RL is that it is also combinable with systemic and topical therapies. Clinical translation for at-home use of LED-RL phototherapy could occur quickly since commercially available FDA-cleared LED-RL devices exist. These features support the clinical use of red light as a promising treatment modality that is convenient, unlikely to cause systemic adverse events, and associated with minimal downtime. LED-RL phototherapy may represent an important advance in light-based treatment modalities because it is non-invasive, painless, inexpensive, portable, and easily combinable with other therapies.

Currently, the safety of red-light therapies is uncertain because of inadequate reporting of adverse events, such as blistering [18]. Furthermore, limited clinical evidence exists pertaining to the safety of LED-RL in different skin types. Fitzpatrick skin phenotypes constitute a method to categorize skin on the basis of its reaction to sun or UV light exposure, ranging from type I (always burn, never tan) to type VI (never burn, always tan) [19]. Fitzpatrick skin types based on subjective self-reporting or physician assessment have flaws due to the unreliability of patient recall regarding burning and tanning [20]. Objective determination of melanin index through reflectance spectrophotometry is most reliable for assessing skin phototype but is costly and burdensome to perform routinely [20]. Ethnicity and race corresponds closely with Fitzpatrick skin types as the majority of Caucasian non-Hispanic individuals are categorized as Fitzpatrick skin types I to III whereas most ethnic skin of color individuals are considered to have Fitzpatrick skin types IV to VI [20]. Although visible light irradiation induced immediate, sustained pigmentation in skin types IV to VI in previous studies, no pigmentation was induced in skin type II at the same doses [6]. Since it is well known that cutaneous response to UV radiation and potentially visible light differs among individuals with different skin types, it is imperative to understand how different skin types respond differently to LED-RL [21].

We previously investigated the safety profile of LED-RL phototherapy at fluences of 160, 320, and 480 J/cm^2^ in all skin types in a dose-escalation, phase I RCT (ClinicalTrials.gov Identifier: NCT02630303) (unpublished data). The study protocol was published in *Trials* [22]. The maximum recommended starting dose (MRSD) of 160 J/cm^2^ was based on maximum LED-RL doses used in previous clinical studies that established safety without adverse events [23, 24]. Starting from the MRSD of 160 J/cm^2^, the dose was escalated in subsequent groups by an algebraic series as described by Spilker: starting with dose (X) increased by an equal amount (in this instance, X = 160 J/cm^2^, 2X = 320 J/cm^2^, 3X = 480 J/cm^2^, and 4X = 640 J/cm^2^) [25]. The maximum tolerated dose (MTD) of HF-LED-RL in skin of color individuals was previously determined to be 320 J/cm^2^, based on one dose-limiting toxicity (DLT) of a 5-mm blister attributed to the LED-RL study device in an African-American male subject after one session of HF-LED-RL at a fluence of 480 J/cm^2^ (unpublished data). The other two Caucasian non-Hispanic subjects also treated with one session of HF-LED-RL at a fluence of 480 J/cm^2^ had no DLTs. The MTD of 320 J/cm^2^ was investigated in a large cohort of 50 subjects, including skin of color individuals. In this large cohort, one Caucasian subject had painless erythema that resolved within 24 to 48 h, and 10 skin of color subjects had transient post-inflammatory hyperpigmentation.

To the best of our knowledge, no clinical trials have been performed to determine the safety of higher doses of HF-LED-RL in Caucasian non-Hispanic individuals. Therefore, the aim of this study is to investigate the safety of HF-LED-RL at doses of 480 and 640 J/cm^2^ in healthy Caucasian non-Hispanic subjects. We hypothesize, based on the findings of our previous trial, that HF-LED-RL phototherapy at 480 and 640 J/cm^2^ will be safe in Caucasian non-Hispanic subjects.

## Methods/Design

### Objectives

The primary objective is to determine the MTD or achieve the predefined study endpoint of 640 J/cm^2^. The secondary objective is to investigate the frequency of adverse events.

### Study design and dose-escalation protocol

This is a single-blind, dose-escalation, randomized, controlled, phase I clinical trial to evaluate the safety of HF-LED-RL phototherapy doses of 480 and 640 J/cm^2^ in Caucasian non-Hispanic subjects. The fluences of 480 and 640 J/cm^2^ are based on the aforementioned Spilker’s dose-escalation protocol (X = 160 J/cm^2^ or MRSD, 2X = 320 J/cm^2^, 3X = 480 J/cm^2^, and 4X = 640 J/cm^2^) [25]. Since the previous phase I RCT for LED-RL phototherapy demonstrated clinical safety of 320 J/cm^2^ in all skin types, the starting dose for this phase I study will be 480 J/cm^2^. The study endpoint of 640 J/cm^2^ was chosen for practicality, as this dose corresponds to 2 h of HF-LED-RL phototherapy at a power density of 872 W/m^2^ and feedback from potential subjects indicated that decreased adherence will likely occur with study procedure durations longer than 2 h. Additionally, a 640 J/cm^2^ dose of HF-LED-RL demonstrated increased anti-fibrotic properties *in vitro* compared with lower doses [26].

**Fig. 1:**
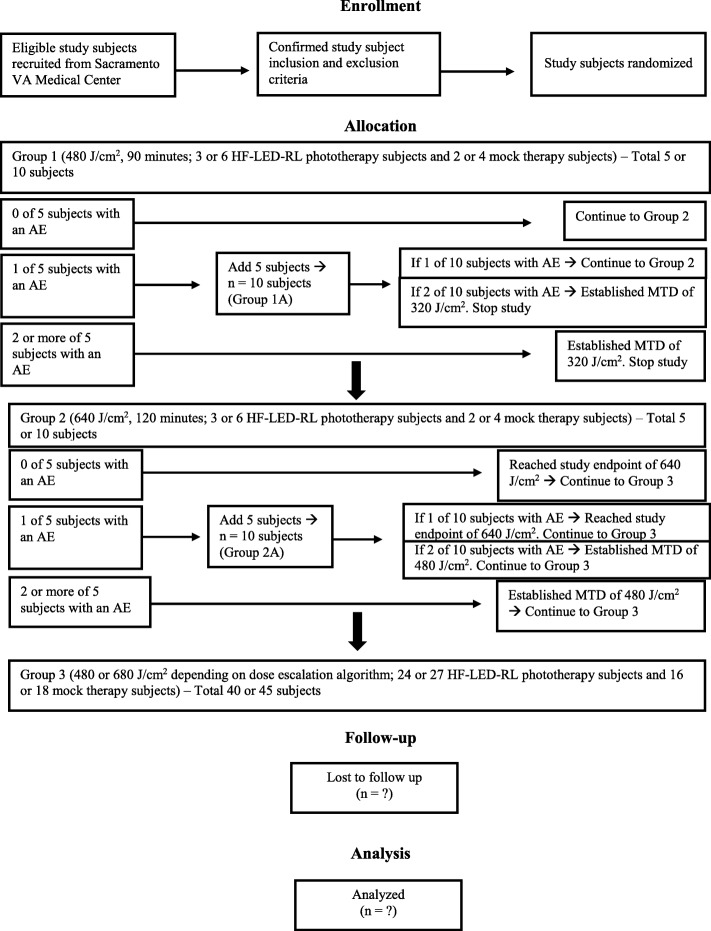
Study dose-escalation flow diagram. The maximum tolerated dose (MTD) is defined as the dose level below the dose producing a dose-limiting toxicity in two or more subjects. Abbreviations: *AE* adverse event, *HF-LED-RL* High-fluence light-emitting diode-red light, *VA* Veterans Affairs.

The dose-escalation protocol is based on the conventional 3 + 3 dose-escalation design to determine the MTD [27]. The 3 + 3 design was chosen because it remains a prevailing method for phase I studies and importantly requires no modeling of the dose–toxicity curve beyond the assumption that toxicity increases with dose. The study dose-escalation flow diagram is presented in Fig. 1. Dose escalation starts at a fluence of 480 J/cm^2^ because this is the recommended starting dose based on the previous phase I study that demonstrated safety of HF-LED-RL at 320 J/cm^2^ (unpublished data). Dose escalation increases to a fluence of 640 J/cm^2^ until at least two subjects among a test-dose cohort experience a DLT (i.e., >20% of subjects with a DLT at that dose level). DLTs are adverse events related to the procedure at the treatment site and are defined as first-degree or higher skin burning or blistering, erythema lasting more than 24 h, edema, pain, ulceration, infection, change in sensation, or muscle weakness or a combination of these. Five more subjects will be enrolled at the same dose level if one DLT is encountered in the first group of five subjects. Common anticipated procedure adverse events are mild and are expected to last less than 24 h and include warmth, erythema, and edema. The MTD is defined as the dose level below the dose at which two or more subjects (>20% of the cohort) experience a DLT.

HF-LED-RL phototherapy and mock therapy at fluences of 480 or 640 J/cm^2^ will be administered to up to 60 Caucasian non-Hispanic subjects. (The total number of subjects will depend on the absence or occurrence of DLTs at each fluence level according to the dose-escalation design.) Subjects will be randomly assigned to groups of five: three subjects are randomly assigned to HF-LED-RL phototherapy and two subjects are randomly assigned to mock therapy. Subjects in group 1 will receive HF-LED-RL or mock irradiation with a treatment time duration of 90 min, equivalent to the starting dose of 480 J/cm^2^. The dose will be escalated in the subsequent group (group 2) to 640 J/cm^2^, associated with a treatment time duration of 120 min. If one DLT is encountered with HF-LED-RL or mock therapy in group 1 or 2, we will repeat the same dose in a new cohort of five additional subjects (group 1A or 2A, respectively).

After either the MTD is established or the study endpoint of 640 J/cm^2^ is achieved, additional HF-LED-RL phototherapy subjects (to reach *n* = 30) and mock therapy subjects (to reach *n* = 20) will receive treatment at that fluence level (group 3). This larger cohort serves to ensure that, based on Hanley’s rule of three, it can be concluded with 95% confidence that fewer than 1 in 10 persons will experience a DLT [28]. If there are no DLTs in the initial group of 480 or 640 J/cm^2^, then an additional 27 HF-LED-RL phototherapy subjects and 18 mock therapy subjects will be enrolled in the large cohort (group 3). If there is a DLT in the initial group of 480 or 640 J/cm^2^ requiring five more subjects to be enrolled in group 1A or 2A, then an additional 24 HF-LED-RL phototherapy subjects and 16 mock therapy subjects will be enrolled in the large cohort (group 3). If the MTD is 320 J/cm^2^, no further testing of this dose will be indicated since the MTD of 320 J/cm^2^ was investigated in 50 subjects with diverse skin types in our previous phase I RCT. Of the large cohort (group 3), the study will be halted if adverse events determined to be device-related reach at least 30% at the review by the quarterly data safety monitoring board (DSMB), which consists of three board-certified dermatologists.

Irradiation will be administered three times per week for three consecutive weeks, which is a standard regimen based on established phototherapy guidelines [29, 30]. Subjects will be recruited from the Sacramento Veterans Affairs (VA) Medical Center campus via flyers and physician referral. Each subject will be compensated weekly, and subjects will receive a prorated amount in the event of study withdrawal. A filled Standard Protocol Items: Recommendations for Interventional Trials (SPIRIT) checklist is available (Additional file 1).

### Subject population

Inclusion criteria and exclusion criteria are summarized in Table 1. Race and ethnicity categories are based on the National Institutes of Health definitions: Caucasian or white is defined as a person with origins from Europe, the Middle East, or North Africa [31]. Hispanic or Latino is defined as a person with origins from Cuba, Mexico, Puerto Rico, or South or Central America [31]. In accordance with manufacturer user guide instructions, all subjects will undergo a 20-min fluence of 106.7 J/cm^2^ of LED-RL photosensitivity testing on his or her non-dominant upper volar forearm with an evaluation 24 h afterwards. Photosensitivity criteria include warmth, erythema, edema, rash, pain, or discomfort lasting more than 24 h at the treatment site.

**Table 1:** Study inclusion and exclusion criteria

Inclusion criteria	
• Healthy subjects of any sex and age	
• Fitzpatrick skin types I, II, or III (non-Hispanic, Caucasian ethnicity)	
• Non-dominant proximal anterior forearm is wide enough to ensure reproducible placement of light-emitting diode-red light (LED-RL) phototherapy or mock therapy hand-held unit	
• Available and willing to attend all clinic visits	
• Able and willing to give informed consent	
Exclusion criteria	
• Subjects with Fitzpatrick skin types IV, V, or VI (ethnic groups)	
• Subjects on any photosensitizing medications (e.g., lithium, phenothiazine antipsychotics, and tetracycline antibiotics)	
• Subjects with light-sensitive conditions	
• Subjects with diabetes mellitus	
• Subjects with a history of melanoma and non-melanoma skin cancer	
• Subjects with systemic lupus erythematous	
• Subjects with open wounds on the non-dominant proximal anterior forearm	
• Subjects with fibrotic skin disease or other skin conditions on the non-dominant proximal anterior forearm	
• Subjects with tattoos that cover the procedure site on the non-dominant proximal anterior forearm	
• Subjects who previously participated in the phase 1 study of LED-RL in human skin (ClinicalTrials.gov Identifier: NCT02630303)	

### Specifications of LED-RL phototherapy and mock therapy hand-held unit

The hand-held LED-RL phototherapy unit (Omnilux New-U, Photo Therapeutics, Carlsbad, CA, USA) is FDA-cleared for treatment of periorbital rhytides at fluences up to 160 J/cm^2^ [32]. The LED-RL unit has a 4.7-by-6.1 cm rectangular aperture and emits visible red light (633 ± 30 nm). To create a standardized procedure time, the procedure duration was based on the average power density of 872 W/m^2^ at a distance of 5 mm from the target surface. Each device tested has a minor variance of 5% in power output per treatment session. The mock therapy unit (Photo Therapeutics) is identical to the LED-RL device but with the LED light disabled. This mock therapy device serves as a temperature-matched control that generates heat comparable to the heat emitted by the LED-RL device, which is less than 2 °C of additional heat, and does not emit red light.

### Study procedure

As previously described [22], the study procedure for subjects receiving LED-RL phototherapy and for subjects receiving mock therapy is identical with the exception of using different devices. The subject’s non-dominant proximal volar forearm will be cleaned with 70% isopropyl alcohol prep pads 1 min prior to device placement. A surgical marking pen will be used to mark three points to outline the procedure area at the start and completion of every session to ensure reproducible placement of the device. The LED-RL phototherapy or mock therapy hand-held unit will be held in place about 5 mm above the clean area by using a tubular elastic retainer net and gauze. The device is at a fixed distance from the skin and not in direct contact with skin in order to minimize direct thermal effects and isolate red light photobiomodulatory effects. Treatment durations for 480 and 640 J/cm^2^ will be 90 and 120 min, respectively. All subjects will be provided with protective eyewear at each session in accordance with the device manufacturer’s user guide. All devices and protective eyewear will be cleaned with anti-germicidal wipes before and after each procedure. The researchers will observe the procedure and assess for any safety issues during and immediately after the procedure. Photographs will be taken pre- and post-procedure at each study visit to record common anticipated procedure adverse events and to ensure uniformity of procedure location at every clinic visit. All participants will be called at the beginning of the week for study visit reminders.

### Safety assessment

**Fig. 2:**
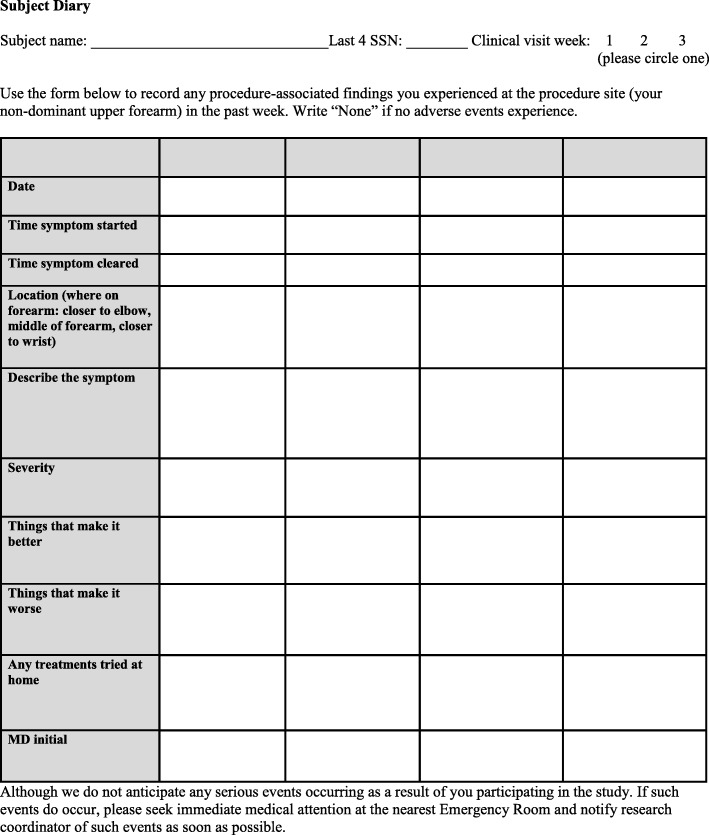
Subject diary template to capture patient-reported outcome

Safety measurements include a subject diary, clinical and photographic assessment for signs and symptoms of adverse events, and physical examination of the treated forearm. All subjects will describe any adverse events in a templated paper diary about every 24 h post-procedure for the duration of study participation (Fig. 2). The subject diary is used to collect device-related symptoms and adverse events that may occur outside of the study visit setting. Subjects with a DLT or adverse event will receive standard medical care. Ancillary and post-study care is available at the Dermatology Clinic at Sacramento VA Medical Center, and no additional compensation will be provided to those who suffer harm from study participation. An independent DSMB will convene quarterly to review and assess any study safety issues. The principal investigator has access to interim results and may make the decision to discontinue a subject from study participation for safety reasons.

### Randomization

Randomization into study groups and interventions will be performed by research coordinators using computer-generated random numbers via the www.randomizer.org website. Ten subjects will be randomly assigned to group 1 (480 J/cm^2^), 10 subjects will be randomly assigned to group 2 (640 J/cm^2^), and 40 subjects will be randomly assigned to group 3 (640 J/cm^2^ or the MTD). If there are no DLTs in the first five subjects of either group 1 or 2, then the remaining five subjects of the 10 subjects randomly assigned to that group will default into group 3. Within each group of five, three subjects will be randomly assigned to the experimental HF-LED-RL phototherapy and two subjects will be randomly assigned to the control intervention. Potential comorbidities that could affect study outcomes were minimized through exclusion criteria of skin cancer, active skin disease in the procedure area, diabetes mellitus, systemic lupus erythematosus, and photosensitivity.

### Blinding

This is a single-blind study, and trial participants will be blinded to the procedure allocation and other subjects’ procedure allocations. The research team will be aware of the randomization since the study coordinators will conduct the treatment sessions and must be able to distinguish between the LED-RL device and mock therapy device to perform the intervention.

### Time frame

**Fig. 3:**
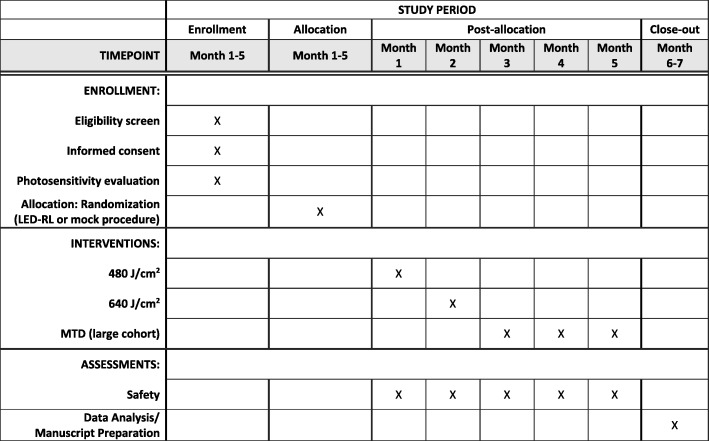
Standard Protocol Items: Recommendations for Interventional Trials (SPIRIT) Figure. Time schedule of enrollment, interventions, and assessments

This study is designed to conclude in 7 months, which includes subject recruitment, performing study procedures, and data analysis. The SPIRIT timeline is available (Fig. 3).

### Statistical analysis

Statistical analysis will be performed by using SAS version 9.4 (SAS Institute Inc., Cary, NC, USA) or similar. Summary statistics of subjects’ baseline characteristics and adverse events will be recorded. All patients who met eligibility criteria and received any treatment during the trial were included in an intention-to-treat analysis. Student’s *t* test or Wilcoxon rank-sum test as appropriate will be used to compare resolution of erythema. A linear mixed-effects model will be used to model the trend of duration of erythema over nine sessions. Chi-squared test or Fisher’s exact test will be used to compare frequency of adverse events between the HF-LED-RL phototherapy intervention group and the mock therapy group. Subgroup analysis will be performed to identify potential differential effects with respect to gender and age. Age subgroup consists of less than 65 or more than 65 years on the basis of published evidence that fibroblasts from older (≥65) individuals have an increased proportion of senescent fibroblasts compared with young (<25) individuals [33]. All tests are two-sided, and *P* values of not more than 0.05 are considered statistically significant. Data analysis relating to protocol non-adherence and any missing data will be discussed with the University of California Davis (UC Davis) Clinical and Translational Science Center (CTSC) Biostatistics service.

### Data collection and dissemination

The PI and clinical research coordinators will have access to the final dataset. Storage of research-related paper files will be in a locked cabinet in the Dermatology Clinic, and all electronic files will be stored on a secured VA research server. No personal identifiable information will be shared outside of the VA.

The full study protocol is made public and available in *Trials.* The research team will communicate trial findings using de-identified study information via publication in a peer-reviewed journal, and assistance of professional writers is not anticipated. Granting public access to the dataset and statistical analysis is not intended.

## Discussion

This follow-up investigator-initiated study aims to provide important knowledge about the safety and cutaneous effects of HF-LED-RL phototherapy at 480 and 640 J/cm^2^ specifically in healthy Caucasian non-Hispanic subjects. Study findings of the dose producing erythema or increased melanogenesis may have clinical implications for preventing hyperpigmentation and supporting photoprotection with use of UV-visible light blockers, such as zinc oxide, iron oxide, or titanium oxide broad-spectrum sunscreens [34]. The importance of this clinical trial is that it may determine stratified dosing based on race and ethnicity that will parallel current UV phototherapy regimens. Furthermore, meta-analysis of the two phase I HF-LED-RL RCTs may provide robust evidence of a differential safety effect of LED-RL because of race and ethnicity. Thus, results obtained from this study may be pivotal in establishing new treatment paradigms and the safety profile for LED-RL based on race and ethnicity that may positively impact clinical practice.

It is hypothesized that skin of color individuals will be more photosensitive to visible red light than Caucasian non-Hispanic subjects, as is evident by a DLT in an African-American male at 480 J/cm^2^ HF-LED-RL in the previous LED-RL phase I study (unpublished data). This is significant to the scientific and medical community because ethnic skin may respond to visible red light differently than to UV light. Darker-pigmented skin is photoprotective for UV wavelengths but is more photosensitive to HF-LED-RL than Caucasian non-Hispanic lightly pigmented skin. In addition to the results of the previous phase 1 study, further evidence for this hypothesis includes clinical observations when using visible light lasers in patients with skin of color [6]. The response of darker skin to LED-RL is analogous to how patients with darker skin are more photosensitive to visible laser light. This disparity in cutaneous effects with visible versus UV light is hypothesized to stem from increased melanin in skin, which has an active absorption spectrum in the visible light region [5]. Although diverse skin types have the same amount of melanocytes, the distribution, shape, and activity of melanosomes vary [35]. Light skin has a less dispersed distribution of melanosomes and decreased melanocyte activity, whereas darker, ethnic skin contains larger and less concentrated melanosomes [35]. Absorption of visible light by melanin generates heat, thereby resulting in deep dermal vasodilatation observed as clinically evident erythema or blister formation [6]. Therefore, this hypothesis corresponds to the selective photothermolysis theory, which highlights the principle of visible light absorption by chromophores in the skin as the basis for laser- and light-based therapies [36].

There are several strengths and improvements in study design compared with the previous phase I study. This phase I study was designed with consultation from the UC Davis CTSC Biostatistics and Bioethical services. In addition to the randomization, mock control, and single blinding, the study’s dose-escalation design increases the robustness of the study and accounts for a possible outlier effect by adding five new subjects at the same dose level if a DLT occurs. Additionally, this study being performed at the Sacramento VA Medical Center is ideal as Caucasian non-Hispanic is the predominant group within the veteran population [37]. Moreover, use of a structured daily study diary with a short recall period of 24 h will allow analysis of meaningful patient-reported outcomes (PROs) and decrease the recall bias of study participants. This study highlights the use of a PRO measure to quantify the frequency and severity of adverse events as a safety endpoint [38]. Implementing a PRO instrument that incorporates the subject’s experience and procedure response can provide valuable evidence in this research study. Verification of PROs will occur through objective clinical examination and photographs at each visit. The DSMB will consist of three board-certified dermatologists with expertise in dermatology, wound healing, and clinical trial methodology to ensure subject safety. To minimize bias, one dermatologist is from the same VA institution and the other two dermatologists are from the university affiliate.

There may be several potential limitations of this study, as previously mentioned [22]. There may be a gender bias toward male subjects recruited within the veteran population since females represent only 9% of veterans nationwide [37]. As males have increased skin collagen and thickness compared with females [39], the penetration of LED-RL may be affected and the MTD may vary by gender. Additionally, there may be a bias toward middle-aged and elderly subjects in the veteran population. Increased age is associated with reduced skin collagen [40] and may require lower doses of LED-RL to produce a DLT, resulting in different MTDs among diverse age groups. Consequently, the MTD obtained from this study may be representative for the sampled population only. Randomization and recruitment of subjects of any sex and age will reduce the risk of bias. Owing to the predominance of older males in the veteran population, block randomization stratified by variables of gender or age is not optimal in the VA setting. In addition, the limitations of the device include that each individual LED-RL device may have different power densities, actual time exposure may vary by a few seconds, and distance of the device from the subject’s skin may differ by a few millimeters. There will be variations of power densities across manufacturers, and the energy output will decrease as devices are used. However, the research team will attempt to minimize variations by assessing the device output using a photometer, using a timer to record light exposure duration, and measuring the distance of the device from the patient’s skin at each session.

Safety information obtained from this study may facilitate future phase II, III, and IV clinical trials of HF-LED-RL phototherapy for skin diseases and conditions. Following a demonstration of safety of HF-LED-RL phototherapy in diverse skin types, our research group may use the results as a basis for stratified dosing based on skin pigmentation in a randomized, split-face, phase II controlled trial to evaluate the efficacy of HF-LED-RL phototherapy in preventing or limiting post-operative scar formation.

## Trial status

Patient recruitment began in February 2018 at the Sacramento VA Medical Center (Mather, CA, USA), and the trial was completed in June 2018.

## Additional file

Additional file 1: Standard Protocol Items: Recommendations for Interventional Trials (SPIRIT) 2013 Checklist. (DOC 122 kb)

## Abbreviations

CTSC: Clinical and Translational Science Center
DLT: Dose-limiting toxicity
DSMB: Data safety monitoring board
FDA: US Food and Drug Administration
HF-LED-RL: High-fluence light-emitting diode-red light
LED: Light-emitting diode
LED-RL: Light-emitting diode-red light
MRSD: Maximum recommended starting dose
MTD: Maximum tolerated dose
PRO: Patient-reported outcome
RCT: Randomized controlled trial
SPIRIT: Standard Protocol Items: Recommendations for Interventional Trials
UC Davis: University of California at Davis
UV: Ultraviolet
VA: Veterans Affairs
